# Better survival for patients with colon cancer operated on by specialized colorectal surgeons – a nationwide population‐based study in Sweden 2007–2010

**DOI:** 10.1111/codi.14760

**Published:** 2019-07-30

**Authors:** M. Bergvall, S. Skullman, K. Kodeda, P.‐A. Larsson

**Affiliations:** ^1^ Department of Surgery Skaraborgs Sjukhus Research Centre Skaraborgs Sjukhus Skövde Skövde Sweden; ^2^ Department of Surgery Taranaki Base Hospital New Plymouth New Zealand; ^3^ Sahlgrenska Academy Gothenburg University Gothenburg Sweden; ^4^ Department of Surgery Skaraborgs Sjukhus Skövde Skövde Sweden; ^5^ Department of Clinical Sciences Lund University Lund Sweden

**Keywords:** Colon cancer, surgery, operation, survival, specialty competence

## Abstract

**Aim:**

Mortality and complication rates after surgery for colon cancer are high, especially after emergency procedures. The aim of the present study was to evaluate the importance of the formal competence of surgeons for survival and morbidity.

**Method:**

The Swedish Colorectal Cancer Registry prospectively records data on patients diagnosed with cancer within the colon and rectum. A cohort of patients operated on for colon cancer between 2007 and 2010 were followed 5 years after surgery. Data on postoperative morbidity, mortality and long‐term survival were compared with regard to formal competency of the most senior surgeon attending the procedure.

**Results:**

This analysis includes 13 365 patients operated on for colon cancer, including 10 434 elective procedures and 2931 emergency cases. The overall 5‐year survival was higher for those operated on by subspecialist colorectal surgeons compared with general surgeons (60% *vs* 48%; *P* < 0.001). Five‐year survival after elective surgery was 63% *vs* 55% (*P* < 0.001) and 35% *vs* 31% (*P* < 0.05) after emergency procedures when performed by colorectal surgeons compared with general surgeons. Postoperative 30‐day mortality was 3% after surgery performed by colorectal surgeons compared with 7% when performed by general surgeons. Mortality at 90 days was 6% after surgery performed by colorectal surgeons compared with 11% for patients operated on by general surgeons (*P* < 0.001).

**Conclusion:**

Subspecialization in colorectal surgery is associated with better outcome for patients operated on for colon cancer, and effort should be made to increase the availability of colorectal surgeons for both acute and elective colon cancer surgery.

**What does this paper add to the literature?**

The present literature on outcomes after colon cancer treatment in the emergency setting is disparate. We present data from a nationwide population‐based registry illustrating that patients with colon cancer operated on by specialist colorectal surgeons have better short‐ and long‐term outcomes, including survival, than patients operated on by general surgeons.

## Introduction

Colon cancer is ubiquitous and a major cause of cancer‐related death. Surgical resection with regional lymphadenectomy is the mainstay of treatment, and approximately 85% of patients are candidates to undergo surgery with curative intent. A high proportion of patients are first diagnosed during an emergency department presentation due to symptomatic disease, and 20% of the procedures are performed as emergencies. The most common indication for emergency or urgent surgery in colon cancer is bowel obstruction, which accounts for some 80% of emergency procedures. Other, less common, causes for an acute operation are intestinal perforation and bleeding 1, 2, 3.

Previous studies have shown lower survival in both the short and long term for patients who require emergency surgery compared with those operated on in the elective setting 2, 3, 4, 5. There are several possible explanations for this. Patients treated acutely are older and frequently suffer from comorbidities, making them more prone to postoperative complications. This may be related to a higher risk of cancer recurrence but also by itself to a reduced overall survival. The risk of dying within 30 days after emergency colon resection is five times higher compared with those patients whose operations are on a scheduled basis. The difference remains after 90 days, when the mortality is four times higher in the acute compared with the elective setting. This applies regardless of whether one corrects for tumour stage and age 2, 5. However, recent publications have presented data showing that outcome is not affected by acute or elective operation if the data are adjusted for the above‐mentioned risk factors 4.

Both emergency procedures and scheduled elective operations are carried out by general surgeons or surgeons specialized in colorectal surgery, depending on the staffing structure in the hospital concerned. There are studies indicating that the influence of surgeon subspecialization and not only the volume of surgery in colon cancer surgery can be significant for survival in both the long and short term 6. However, most studies involve the entire group of patients undergoing colorectal cancer surgery and do not distinguish between colon and rectal cancer 6, 7, 8, 9. In a UK study looking at short‐ and long‐term survival for colon cancer patients undergoing elective surgery, a higher 5‐year survival was found for patients operated on by a specialist colorectal surgeon. The authors concluded that the lower postoperative mortality associated with operations performed by specialized colorectal surgeons was the main reason for better survival 10. A US study comparing high‐ and low‐volume centres reported higher postoperative survival in the former, which was explained by the fact that in the high‐volume centres there was a better ability to detect and treat complications 11. Whether formal surgical competence and volume are important for outcome is not easily resolved by randomized trials, but population‐based registers can be a possible source of information to shed light on this issue. Since 2007, all newly discovered cases of colon cancer in Sweden are registered in the national Swedish ColoRectal Cancer Registry (SCRCR). This database covers 99% of all colonic adenocarcinomas in Sweden 12. The main objective of the registry is quality control. However, the prospective accrual of a large number of data from virtually all newly diagnosed patients with colorectal cancer in a defined population renders it suitable for studies regarding parameters that are not easily studied in randomized studies, such as survival difference after elective and emergency surgery or the influence of surgical competence on survival.

The purpose of this study was therefore to evaluate the impact of the surgeon's formal competence on short‐ and long‐term results in colon cancer surgery.

## Method

This is a retrospective cohort study of all patients operated on for colon cancer and registered in the SCRCR from 2007 to 2010. The SCRCR is a so‐called quality register and the coverage rate is close to 100% 12. During the study period, 16 397 patients were diagnosed with colon cancer in Sweden. Only adenocarcinomas are included in the registry, but formal histological verification is not required for registration of new cases 12. A tumour situated within 15 cm of the anal verge is reported as rectal cancer and a tumour situated more than 15 cm from the anal verge is reported as colon cancer. Adenocarcinomas of the appendix are included in the registry. The registry excludes carcinoma *in situ* and tumours found at autopsy. The study covers 13 365 patients operated on by resection of the tumour. Of these patients, 12 972 had one tumour at the time of surgery, 344 patients had two tumours, 39 had three tumours and 10 had four tumours. In most cases those who had two or more tumours had synchronous tumours. Only a few had new, metachronal tumours at another diagnostic event. Data from patients operated on for a second metachronous tumour during the study time were only recorded with regard to surgery for their first colon cancer. The SCRCR records data pre‐, peri‐ and postoperatively. We studied 32 parameters from the registry (Table 1) and compared patients who were operated on in the emergency setting with those undergoing scheduled procedures. Postoperative complications are included in the registry only if they occur within the current period of care of the primary operation or within 30 days of surgery 12. Patients operated on for a primary colon cancer were included in the registry, and data were analysed regardless of lymph node status, M‐stage, concomitant intestinal diseases or if the procedure was performed with curative or palliative intent.

**Table 1:** Registered parameters, extracted from the Swedish ColoRectal Cancer Registry.

Age
Gender
Date of diagnosis
Date for surgery
Preoperative staging
Preoperative multidisciplinary team treatment conference
Site of tumour
Covering ileostomy
Permanent stoma
Intestinal perforation during surgery
Locally radical surgery (as reported by surgeon)
Curative surgery (as reported by surgeon)
Indication for emergency surgery
Type of surgery
Attending surgeon's competence level
Time of day for start of surgery
TNM staging
Number of nodes in specimen
Microscopically radical surgery (according to pathology report)
Circumferential resection margin
American Society of Anesthesiologists classification
Postoperative complications
Intensive care
Reoperations
Length of stay in hospital
Unscheduled readmittance to hospital ward
Mortality at 30 days
Mortality at 90 days
Cause of death
Adjuvant chemotherapy
Palliative chemotherapy
Survival

The division into acute and elective procedures was performed according to specific criteria and recorded in accordance with the SCRCR 12. In short, a cancer resection was considered as ‘acute’ when the operation was medically indicated to be performed acutely, even if the actual operation was done in an ‘elective list’. This means that urgent procedures have also been classified as emergencies in the present report. The elective procedures were all performed as planned procedures and all these patients were operated on after diagnostic workup and preoperative procedures according to normal Swedish standards of care.

The oncological result of resections was reported both as a subjective impression of the surgeon immediately after the procedure and a systematic microscopic analysis of tumour, resection margins and lymph nodes in the pathology report. The term locally radical surgery refers to the surgeon's impression of resection regardless of M‐stage, ‘curative operation’ refers to the surgeon's impression of a locally radical procedure in M0 patients (Tables 1 and 2). Data on these two parameters are entered as checkbox choices by the surgeons as either yes, no or doubtful. Data have been analysed as radical, yes or no and the cases classified by the surgeons as doubtful have been counted as no, not radical, in the multivariate analysis. The term radical resection refers to a specimen with microscopically free resection margins regardless of M‐stage and curative resection means R0 resection in an M0 patient (Tables 1 and 3 ). Cases presented as doubtful have a missing pathology report or a high‐quality contrast‐enhanced thoracoabdominal CT scan for evaluation of M‐stage. Data on this parameter have been included in the multivariate analysis radical, yes or no and cases reported as doubtful in the registry have been classified as not radical resection.

**Table 2:** Data extracted from the Swedish ColoRectal Cancer Registry with regard to emergency procedures and electively operated patients. Differences for all presented parameters are statistically significant (*P* < 0.001).

	Elective (*n* = 10 434) (%)	Emergency (*n* = 2931) (%)	Total (*n* = 13 365) (%)
Preoperative multidisciplinary conference			
Yes	70.0	7.6	63.8
No	29.7	92.3	35.9
Missing data	0.3	0.1	0.3
Location of tumour			
Right colon and hepatic flexure	48.0	43.0	46.8
Transverse colon	9.0	10.5	9.3
Left colon and sigmoid	42.9	46.2	43.6
Inconclusive	0.1	32.2	16.3
Specialist colorectal surgeon attending			
Yes	88.2	67.8	83.7
No	11.8	32.2	16.3
Bowel perforation during surgery			
Yes	1.8	6.0	2.7
No	97.4	92.7	96.4
Missing data	0.8	1.3	0.9
Locally radical surgery			
Yes	90.1	68.3	85.3
No	4.8	18.8	7.8
Doubtful	4.9	12.5	6.6
Missing data	0.2	0.4	0.3
Curative operation			
Yes	79.4	50.0	73.0
No	11.0	29.2	15.0
Doubtful	9.2	20.2	11.6
Missing data	0.4	0.6	0.4
Permanent stoma			
Yes	6.2	22.8	9.8
No	93.1	76.3	89.4
Missing data	0.8	1.0	0.8
T‐stage			
pT0	0.2	0.0	0.2
pT1	6.2	1.2	5.1
pT2	13.9	4.4	11.8
pT3	60.7	53.5	59.1
pT4	16.9	33.5	20.6
pTx	1.7	6.6	2.8
Missing data	0.3	0.9	0.4
N‐stage			
pN0	57.0	38.7	53.1
pN1	22.7	24.0.	23.0
pN2	17.2	27.5	19.4
Missing data	3.5	10.8	5.1
M‐stage			
M0	86.9	70.4	83.3
M1	12.7	28.8	16.2
Missing data	0.4	0.8	0.5
American Society of Anesthesiologists classification			
1	16.1	12.2	15.2
2	52.3	41.6	50.0
3	26.2	33.1	27.7
4	2.1	6.6	3.1
5	0.0	0.2	0.0
Missing data	3.3	6.4	4.0
Intensive care after surgery			
Yes	6.3	12.6	7.7
No	68.4	60.4	66.7
Missing data	0.3	0.5	0.4

**Table 3:** Multivariate analysis of 30‐day mortality after colon cancer surgery. Odds ratio for risk of death.

	OR	95% CI
Age	1.04	1.042–1.069
Colorectal surgeon	0.73	0.559–0.948
Emergency surgery	2.87	2.268–3.631
Intestinal perforation	1.02	0.641–1.633
Radical resection	0.76	0.589–0.978
Curative resection	0.78	0.545–0.953
American Society of Anesthesiologists classification	2.28	1.952–2.673
T‐stage	1.62	1.383–1.892
Intensive care	5.88	4.572–7.560
Postoperative complication	9.95	7.574–13.077

In the SCRCR, surgeons are classified as accredited colorectal surgeons, colorectal specialists, general surgeons or registrars. Colorectal surgery is not recognized as an official medical speciality and there is no official examination or board certification in colorectal surgery in Sweden. Several surgeons doing colorectal surgery have credentials in coloproctology issued either by the Swedish Society for Colorectal Surgery or UEMS (the European Union of Medical Specialists). Registration in the registry as an accredited colorectal surgeon is based on these credentials and registration as a colorectal specialist is based on self‐reported major occupation in clinical work in combination with a specialist registration in general surgery. In this study, we have defined accredited colorectal surgeons and colorectal specialists as colorectal surgeons. Other noncolorectal specialists and senior registrars were defined as general surgeons. In all cases, we have registered the highest formal competence attending in the operating room with regard to colorectal surgery. It is not possible to analyse SCRCR data with regard to who is performing, assisting at, or supervising the actual operation.

### Statistics

Data were analysed on IBM spss software platform version 25 (IBM‐SPSS, IBM, Armonk, New York, USA). Descriptive statistics, frequencies and proportions are presented in Table 2. Central tendencies for continuous data are presented as mean ± standard deviation. For categorical variables, proportions were compared using the chi‐square test. A multivariate logistic regression analysis was performed to analyse the relationship of the studied parameters with survival at 30 days, 90 days and 5 years. Survival analysis was also performed with the Kaplan–Meier model. Although the SCRCR coverage rate is close to 100%, some data may be missed in reporting individual parameters, and the number of patients may therefore vary in the analyses. Missing data have been omitted in the performed comparisons and the multivariate analysis. Parameters with more than 5% missing data were not analysed further.

### Ethics

The study was approved by the national steering committee of the SCRCR and the regional board for ethics in research, Gothenburg, Sweden (ref. 097‐2014).

## Results

Of the 13 365 operations in this study, 2931 were emergency procedures and 10 434 planned procedures. Of the patients undergoing emergency surgery, 67.8% were operated on by colorectal surgeons and 32.2% were operated on by general surgeons. The corresponding figures for elective operations were 88.2% and 11.8%, respectively (Table 2).

Patients undergoing emergency surgery were on average 72.7 ± 12.4 years old, whereas the mean age of electively operated patients was 71.9 ± 11.1 years. The group of patients treated with emergency surgery also suffered from more concomitant health problems. Of those operated on acutely, 39.9% were in American Society of Anesthesiologists (ASA) class 3–5, compared with 28.3% for patients undergoing elective operations. Tumour stage was more advanced with twice as many T4 tumours in the emergency group, and emergency cases were nearly four times more likely to receive a permanent stoma. In 90.1% of patients undergoing elective operations the tumour was radically removed. The corresponding figure was only 68.3% for patients operated on in the emergency setting. Surgery was considered curative in 79.4% of cases after elective surgery but only 50.0% of the emergency procedures were considered curative.

Postoperative complications occurred in 26.2% of all patients (Table 4), with 33.7% of patients undergoing acute operations and 24.2% of the electively operated patients being affected. Mortality was high in patients stricken by complications. Among acute patients with postoperative complications, 75% died within 5 years compared with 44.2% (*P* < 0.001) of electively operated patients with postoperative complications. Anastomotic dehiscence occurred three times more frequently after acute operations (6.0% compared with 1.8% after elective procedures). Twice as many emergency patients required unscheduled care in an intensive care unit (ICU) after surgery (12.6% *vs* 6.3%). The average number of retrieved lymph nodes in specimens was 17.8 ± 10.3. After elective procedures, the average number of retrieved lymph nodes was 18.1 ± 10.2 compared with 16.8 ± 10.5 after emergency procedures. When the resection was performed by a colorectal surgeon the average number of retrieved nodes was 18.1 ± 10.2 compared with 16.8 ± 10.4 in specimens after resection by a general surgeon.

**Table 4:** Mortality after colon cancer surgery in relation to complications requiring therapy after emergency and planned procedures (number of patients; *P* < 0.001).

Postoperative complication	30‐day mortality (%)		90‐day mortality (%)		5‐year mortality (%)		Sum
	Yes	No	Yes	No	Yes	No	
Emergency procedures							
Yes	235 (24.0)	746 (76.0)	318 (32.4)	663 (67.6)	736 (75.0)	245 (25.0)	981
No	65 (3.4)	1868 (96.6)	196 (10.1)	1737 (89.9)	1135 (58.7)	798 (41.3)	1933
Sum	300 (10.3)	2614 (89.7)	514 (17.6)	2400 (82.4)	1871 (64.2)	1043 (35.8)	2914
Planned procedures							
Yes	188 (7.5)	2322 (92.5)	277 (11.0)	2233 (89.0)	1110 (44.2)	1400 (55.8)	2510
No	31 (0.4)	7851 (99.6)	146 (1.9)	7736 (981)	2540 (32.2)	5342 (67.8)	7882
Sum	219 (2.1)	10 173 (97.9)	423 (4.1)	9969 (95.9)	3650 (35.1)	6742 (64.9)	10 392
All procedures							
Yes	423 (12.1)	3068 (87.9)	595 (17.0)	2896 (83.0)	1846 (52.9)	1645 (47.1)	3491
No	96 (1.0)	9719 (99.0	342 (3.5)	9473 (96.5)	3675 (37.4)	6140 (62.6)	9815
Sum	519 (3.9)	12 787 (96.1)	937 (7.0)	12 369 (93.0)	5521 (41.5)	7785 (58.5)	13 306

When we looked at 30‐ and 90‐day mortality, we found almost twice the survival rate for patients operated on by colorectal surgeons for the entire group of patients (*P* < 0.001; Table 5). Thirty‐day mortality did not differ when we split the patients into acute and elective surgery groups, but for 90‐day mortality we saw a somewhat higher mortality rate for the elective patients operated on by general surgeons compared with colorectal surgeons (5.1% *vs* 3.9%; *P* = 0.05). No statistically significant difference was found for the emergency procedures.

**Table 5:** Mortality after 30 and 90 days for patients operated on by colorectal surgeons and general surgeons, respectively. Difference regarding 30‐ and 90‐day mortality between patients operated on by colorectal or general surgeons were both statistically significant (*P* < 0.001).

	30‐day mortality			90‐day mortality		
	Yes	No	*n*	Yes	No	*n*
Colorectal surgeon	3.4%	96.6%	11 154	6.2%	93.8%	11 154
General surgeon	6.6%	93.4	2175	11.3%	88.7%	2175
Total no. of patients	523	12 806	13 329	941	12 388	13 329

A multivariate analysis was performed to investigate which factors had an impact on survival after 30 days, 90 days and 5 years (Tables 3, 6 and 7). This indicates that colorectal specialization is important for survival in both the short and long term. Mortality for emergency patients was higher: the OR for 30‐day mortality was 2.9 and for 90‐day mortality 2.7. The OR for 5‐year mortality in emergency patients was 2.3. Other factors that were related to increased mortality were: postoperative complications, the need for postoperative ICU care, ASA classification and T‐stage.

**Table 6:** Multivariate analysis of 90‐day mortality after colon cancer surgery. Odds ratio for risk of death.

	OR	95% CI
Age	1.04	1.030–1.048
Colorectal surgeon	0.75	0.615–0.915
Emergency surgery	2.74	2.306–3.253
Intestinal perforation	0.99	0.672–1.451
Radical resection	0.70	0.576–0.847
Curative resection	0.56	0.477–0.651
American Society of Anesthesiologists classification	2.04	1.819–2.297
T‐stage	1.78	1.585–2.003
Intensive care	4.07	3.314–5.008
Postoperative complication	3.62	3.032–4.328

**Table 7:** Multivariate analysis of mortality 5 years after colon cancer surgery. Odds ratio for risk of death.

	OR	95% CI
Age	1.04	1.032–1.040
Colorectal surgeon	0.83	0.737–0.929
Emergency surgery	2.27	2.047–2.519
Intestinal perforation	1.52	1.179–1.969
Radical resection	1.21	1.075–1.355
Curative resection	0.54	0.501–0.589
American Society of Anesthesiologists classification	1.65	1.555–1.758
T‐stage	2.26	2.127–2.402
Intensive care	2.00	1.693–2.356
Postoperative complication	1.35	1.221–1.484

**Figure 1:**
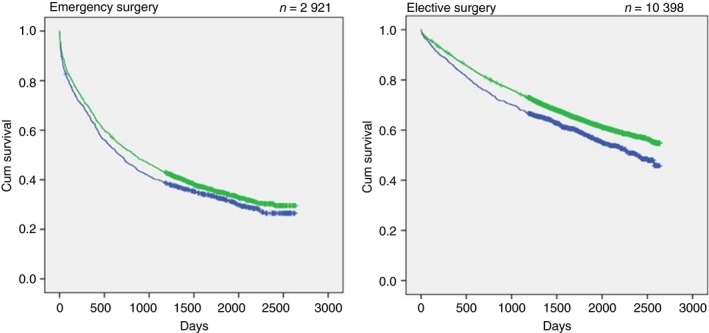
Kaplan–Meier curves showing 5‐year survival after surgery performed by colorectal surgeons (green dots) and general surgeons (blue dots). Overall 5‐year survival was 60% when performed by colorectal surgeons compared with 48% after surgery performed by general surgeons (*P* < 0.001). Corresponding figures after emergency procedures were 36.6% compared with 33.4% (*P* < 0.05) and 65.5% compared with 59.7% after elective surgery (*P* < 0.001). Data are missing for 10 emergency procedures and 36 elective operations.

This study shows an improved 5‐year survival (60%) for patients operated on by colorectal surgeons compared with patients operated on by general surgeons, (48%) (Fig. 1). When we split the data into acute and elective operations, the difference was still significant in both groups (*P* < 0.001; Fig. 1). This difference remained after adjustment for 30‐ and 90‐day mortality for the total patient group and for those patients operated on electively, but not for those who were operated on acutely.

## Discussion

The present study shows a reduced mortality rate for colon cancer patients operated on by colorectal surgeons in the short term, and an improved 5‐year survival after both acute and elective surgery. The significance of colorectal subspecialization for survival also seems to persist when adjusted for confounding factors. The fact that colorectal subspecialization seems to be significant for survival is supported by previous studies, although most include all patients operated on for colorectal cancer and not only colon cancer. In a Spanish study of 1046 patients operated on for colorectal cancer between 1993 and 2006 and comparing colorectal surgery performed by colorectal surgeons with general surgeons, the number of stoma creations and frequency of anastomotic dehiscence were lower. Postoperative mortality was also lower when surgery was performed by a colorectal surgeon 6. These data are also supported by studies from Australia and the UK 7, 8. The limitation of the present study is its design as a retrospective cohort study, and the observed survival benefit does not prove a causal relationship between surgical specialization and survival.

A British multicentre study following 1856 patients in 16 hospitals after elective colon cancer surgery between 2001 and 2004 showed lower postoperative mortality (4.5% *vs* 7.0%) and better 5‐year survival in patients operated on by a colorectal surgeon (72.2% *vs* 65.6%). The authors concluded that the reason for better survival after surgery was a lower proportion of postoperative complications when surgery was performed by a colorectal surgeon 10. These data are in line with the results of the present report. Short‐term and 5‐year mortality in the present study were lower when emergency procedures were performed by colorectal surgeons. However, the difference in 5‐year survival in emergency cases operated on by colorectal and general surgeons was not statistically significant when adjusted for 30‐ and 90‐day mortality,

Our data on lower 30‐ and 90‐day mortality after emergency colon cancer surgery when performed by colorectal surgeons differ from those of a recent Danish national register study where no statistically significant difference in 90‐day mortality after open emergency colorectal cancer procedures was observed 13. The Danish study was performed on data from patients operated on between 2005 and 2015 and reports higher overall mortality at 30 and 90 days (16% and 25%, respectively) compared with Swedish data from 2007 to 2011 (9.5% and 16.6%, respectively) 12. However, the Danish study also included patients with rectal cancer 13.

Factors other than surgical proficiency and expertise have been suggested as explanations for better results after colorectal surgery. A US study found that patients having colon cancer surgery in high‐volume centres had a lower postoperative mortality rate within 30 days than patients in low‐volume centres 11. These authors were unable to prove that high‐volume surgeons had better survival. The better survival rate in high‐volume hospitals was explained by better care and the ability to detect and treat surgical complications 11.

Our results verify previous reports that emergency surgery in colon cancer is related to higher mortality in both the long and the short term 4, 14. A previous analysis of patients with colon cancer subjected to emergency surgery that used risk‐adjusting methods found that the increased mortality could be explained by patient characteristics and concluded that the emergency operation itself was not a risk factor for increased mortality 4. Thus, surgery should not be postponed in these cases but efforts should be made to optimize surgery when necessary.

In a Danish register study, 2157 patients with colon cancer were analysed regarding risk factors for 30‐day mortality. Mortality was 22.1%, and it was found that the strongest cause of early death was medical postoperative complications, for example cardiovascular, renal, thromboembolic and nonsurgical‐site infectious (OR = 11.7). Postoperative surgical complications occurred in one‐fifth of the cases but had no significant effect on mortality 15. As we have not divided complications into medical and surgical, the numbers are difficult to compare, but postoperative complications and mortality are major contributors affecting long‐term survival and the present study adds evidence to support the proposition that specialized surgical training reduces complications.

## Conclusion

Our results indicate that short‐term results and 5‐year survival are improved in colon cancer surgery when performed by specialized colorectal surgeons. This is also pertinent in the emergency setting, and efforts should be made to ensure the availability of specialized colorectal surgeons when such procedures need to be performed.

## Conflicts of interest

There are no conflicts of interest to declare.

## Author contributions

MB has together with the three co‐authors analysed data and written the manuscript. SS has together with the three co‐authors analysed data and written the manuscript. KK has together with the three co‐authors analysed data and written the manuscript. P‐AL has together with the three co‐authors analysed data and written the manuscript.

## References

[1] Bass G , Fleming C , Conneely J , Martin Z , Mealy K . Emergency first presentation of colorectal cancer predicts significantly poorer outcomes: a review of 356 consecutive Irish patients. Dis Colon Rectum 2009; 52: 678–84. 19404074 10.1007/DCR.0b013e3181a1d8c9

[2] McArdle CS , Hole DJ . Emergency presentation of colorectal cancer is associated with poor 5‐year survival. Br J Surg 2004; 91: 605–9. 15122613 10.1002/bjs.4456

[3] Oliphant R , Mansouri D , Nicholson GA *et al* Emergency presentation of node‐negative colorectal cancer treated with curative surgery is associated with poorer short and longer‐term survival. Int J Colorectal Dis 2014; 29: 591–8. 24651957 10.1007/s00384-014-1847-5

[4] Weixler B , Warschkow R , Ramser M *et al* Urgent surgery after emergency presentation for colorectal cancer has no impact on overall and disease‐free survival: a propensity score analysis. BMC Cancer 2016; 16: 208. 26968526 10.1186/s12885-016-2239-8 PMC4787247

[5] van Leeuwen BL , Pahlman L , Gunnarsson U , Sjovall A , Martling A . The effect of age and gender on outcome after treatment for colon carcinoma. A population‐based study in the Uppsala and Stockholm region. Crit Rev Oncol Hematol 2008; 67: 229–36. 18440820 10.1016/j.critrevonc.2008.03.005

[6] Biondo S , Kreisler E , Millan M *et al* Impact of surgical specialization on emergency colorectal surgery outcomes. Arch Surg 2010; 145: 79–86. 20083758 10.1001/archsurg.2009.208

[7] Platell C , Lim D , Tajudeen N , Tan JL , Wong K . Dose surgical sub‐specialization influence survival in patients with colorectal cancer? World J Gastroenterol 2003; 9: 961–4. 12717838 10.3748/wjg.v9.i5.961 PMC4611405

[8] McArdle CS , Hole DJ . Influence of volume and specialization on survival following surgery for colorectal cancer. Br J Surg 2004; 91: 610–7. 15122614 10.1002/bjs.4476

[9] Buurma M , Kroon HM , Reimers MS , Neijenhuis PA . Influence of individual surgeon volume on oncological outcome of colorectal cancer surgery. Int J Surg Oncol. 2015; 2015: 464570. 26425367 10.1155/2015/464570 PMC4573626

[10] Oliphant R , Nicholson GA , Horgan PG *et al* The impact of surgical specialisation on survival following elective colon cancer surgery. Int J Colorectal Dis 2014; 29: 1143–50. 25034593 10.1007/s00384-014-1965-0

[11] Billingsley KG , Morris AM , Dominitz JA *et al* Surgeon and hospital characteristics as predictors of major adverse outcomes following colon cancer surgery: understanding the volume‐outcome relationship. Arch Surg 2007; 142: 23–31. discussion 2. 17224497 10.1001/archsurg.142.1.23

[12] Kodeda K , Nathanaelsson L , Jung B *et al* Population‐based data from the Swedish Colon Cancer Registry. Br J Surg 2013; 100: 1100–7. 23696510 10.1002/bjs.9166

[13] Degett TH , Dalton SO , Christensen J , Sogaard J , Iversen LH , Gogenur I . Mortality after emergency treatment of colorectal cancer and associated risk factors‐a nationwide cohort study. Int J Colorectal Dis 2019; 34: 85–95. 30327873 10.1007/s00384-018-3172-x

[14] Cuffy M , Abir F , Audisio RA , Longo WE . Colorectal cancer presenting as surgical emergencies. Surg Oncol 2004; 13: 149–57. 15572097 10.1016/j.suronc.2004.08.002

[15] Iversen LH , Bulow S , Christensen IJ , Laurberg S , Harling H , Danish Colorectal Cancer Group . Postoperative medical complications are the main cause of early death after emergency surgery for colonic cancer. Br J Surg 2008; 95: 1012–9. 18563787 10.1002/bjs.6114

